# Styrene Trimer May Increase Thyroid Hormone Levels via Down-Regulation of the Aryl Hydrocarbon Receptor (AhR) Target Gene UDP-Glucuronosyltransferase

**DOI:** 10.1289/ehp.10724

**Published:** 2008-02-28

**Authors:** Yukie Yanagiba, Yuki Ito, Osamu Yamanoshita, Shu-Yun Zhang, Gen Watanabe, Kazuyoshi Taya, Chun Mei Li, Yuko Inotsume, Michihiro Kamijima, Frank J. Gonzalez, Tamie Nakajima

**Affiliations:** 1 Department of Occupational and Environmental Health, Nagoya University Graduate School of Medicine, Nagoya, Japan; 2 Department of Biomedical Sciences, College of Life and Health Science, Chubu University, Kasugai, Japan; 3 Department of Basic Veterinary Science, United Graduate School of Veterinary Sciences, Gifu University, Gifu, Japan; 4 Department of Veterinary Medicine, Faculty of Agriculture, Tokyo University of Agriculture and Technology, Tokyo, Japan; 5 National Institute for Environmental Studies, Ibaraki, Japan; 6 Laboratory of Metabolism, National Cancer Institute, Bethesda, Maryland, USA

**Keywords:** aryl hydrocarbon receptor, cytochrome P450 1A, styrene trimer, thyroid hormone, UDP-glucuronosyltransferase

## Abstract

**Background:**

Styrene trimers (STs) are polystyrene-container–eluted materials that are sometimes detected in packaged foods. Although the possible endocrine-disrupting effects of STs, such as estrogenic activities, have been reported, their potential thyroid toxicity, such as that caused by the related endocrine disruptor 2,3,7,8-tetrachlorodibenzo-*p*-dioxin (TCDD), has not been studied in detail.

**Objective:**

Using wild-type and aryl hydrocarbon receptor (*Ahr*)–null mice, we investigated whether 2,4,6-triphenyl-1-hexene (ST-1), an isomer of STs, influences thyroxin (T_4_) levels in the same manner as TCDD, which induces UDP-glucuronosyltransferase (UGT) via the AhR, resulting in a decrease in T_4_ levels in the plasma of mice.

**Methods:**

Both wild-type and *Ahr*-null mice (five mice per group) were treated for 4 days by gavage with ST-1 (0, 32, or 64 μmol/kg).

**Results:**

High-dose (64 μmol/kg) ST-1 decreased the expression of AhR, cytochrome P450 (CYP) 1A1/2, UGT1A1/A6, and CYP2B10 mRNAs and the enzyme activity for CYP1A and UGT1A only in the wild-type mice. This dose decreased AhR DNA binding, but paradoxically increased AhR translocation to the nucleus. In contrast, a high dose of ST-1 increased T_4_ levels in the plasma in wild-type mice but did not influence T_4_ levels in *AhR*-null mice.

**Conclusions:**

Although ST-1 treatment might cause an increase in AhR levels in the nucleus by inhibiting AhR export, this chemical down-regulated AhR mRNA, thus leading to down-regulation of AhR target genes and an increase in plasma T_4_ levels.

Polystyrene resins are widely used for food packaging, such as take-out food containers, coffee cups, meat trays, soup bowls, and salad boxes. From these containers, styrene oligomers sometimes migrate into the food (Sakamoto et al. 2000). With the polystyrene-container–packed instant foods such as noodles and rice, migration from the cups into these foods after cooking resulted in 0–8.1 μg/cup of 2,4,6-triphenyl-1-hexene (ST-1), 0–13.8 μg/cup of 1a-phenyl-4a-(1′-phenylethyl) tetralin (ST-2) and 1a-phenyl-4e-(1′-phenylethyl) tetralin (ST-3), 0–5.3 μg/cup of 1-e-phenyl-4a-(1′-phenylethyl) tetralin (ST-4), and 0–8.4 μg/cup of 1-e-phenyl-4e-(1′-phenylethyl) tetralin (ST-5) (Kawamura et al. 1998). When instant noodles or soups in the polystyrene cups were cooked according to the manufacturer’s instructions, 0.8–21 ng/g ST-1, 1.1–16 ng/g ST-2, 1.2–19 ng/g ST-3, 0.6–8.6 ng/g ST-4, 0.4–8.6 ng/g ST-5, 0.4–5.1 ng/g 1e,3e,5e-triphenylcyclohexane (ST-7), and 0.3–1.3 ng/g structurally uncertain isomer (ST-8) migrated into the food (Kaneko et al. 2003). Some polystyrene instant noodle containers themselves contained 80–980 μg/g ST-1, 10–980 μg/g ST-2, 20–970 μg/g ST-3, 190–650 μg/g ST-4, 210–670 μg/g ST-5, 10–40 μg/g 1e,3e,5a-triphenylcyclohexane (ST-6), 80–280 μg/g ST-7, and 40–90 μg/g ST-8 (Kaneko et al. 2003). However, the contents of styrene trimers (STs) in these polystyrene containers were clearly lower than those reported by Sakamoto et al. (2001), suggesting that the quality of polystyrene food containers is improving.

Although people are often potentially exposed to STs, there are some conflicting findings about their toxicity. A binding assay for several receptors showed that synthesized STs [ST-1, ST-3, and 1-phenyl-4-(2-phenylethyl) tetralin (ST-12)] did not bind to estrogen receptors (ERs), androgen receptors, and thyroid hormone receptors of uterus, ventral prostate, and liver from Sprague-Dawley rats (Date et al. 2002; Ohno et al. 2002a, 2002b). Similarly, at 10^–5^ M, these compounds did not accelerate the proliferation of MCF-7 human breast cancer cells, suggesting that they do not have potentially adverse effects similar to those of estrogen (Nobuhara et al. 1999; Ohno et al. 2002a). In addition, oral administration of oligomers extracted from general-purpose, high-impact, and expandable polystyrene materials did not influence the uterine weight in Wistar rats (Bachmann et al. 1998). Three STs (ST-1, ST-3, and ST-12) from noodle containers did not influence the uterine weight or serum prolactin concentration in of Sprague-Dawley rats (Date et al. 2002; Nobuhara et al. 1999; Ohno et al. 2002a). In contrast, synthesized STs (ST-1, ST-2, ST-3, ST-4, and ST-5) increased the proliferation of human MCF-7 cells at 10^–6^ M (Ohyama et al. 2001), a concentration considerably lower than those used in the studies by Nobuhara et al. (1999) and Ohno et al. (2002a), suggesting that these chemicals may bind human ER-α at a lower concentration. However, the estrogenic activity of STs observed by Ohyama et al. (2001) was extremely weak—1/68,000—which was less than that of 17β-estradiol. More recently, ST-1, ST-3, and ST-4 [0–1,000 μg/kg body weight (bw) per day] were reported to markedly decrease the relative weight of testes in the offspring of Sprague-Dawley rats; ST-3 and ST-4, but not ST-1, decreased the relative weight of brain, whereas ST-1 and ST-4 significantly decreased Sertoli cell counts and plasma luteinizing hormone levels (Ohyama et al. 2007). Conversely, these two STs increased plasma testosterone levels and shortened the anogenital distance in the offspring. In addition, ST-2, ST-3, ST-4, and ST-5 had binding affinities for androgen receptor (Satoh et al. 2001). Thus, although the reason for the above-mentioned conflicting reports has not been resolved, some STs may have potential toxicity as endocrine disruptors.

UDP-glucuronosyltransferase (UGT) is involved in the conjugation of xenobiotics as well as endogenous hormones such as thyroxin (T_4_) and steroids (Emi et al. 1995; Maglich et al. 2004). Two UGT families, UGT1 and UGT2, exist (Burchell and Coughtrie 1997; Mackenzie et al. 1997). In the UGT1A family, UGT1A1 and UGT1A6 catalyze the conjugation of bilirubin and phenols as well as T_4_ (Emi et al. 1995; Nishimura et al. 2005). UGT1A1 is regulated not only by the aryl hydrocarbon receptor (AhR) but also by the constitutive androstane receptor (CAR) (Sugatani et al. 2001, 2004; Zhou et al. 2005), whereas UGT1A6 seems to be regulated only by AhR, because a single dose of 10 μg/kg 2,3,7,8-tetrachlorodibenzo-*p*-dioxin (TCDD) to pregnant mice induced this isozyme only in *AhR* (+/–) offspring, not in *AhR*-null mice (Nishimura et al. 2005). This resulted in a marked reduction of total and free T_4_ levels in the serum of *AhR* (+/–) mice. In addition, 0.003–30 μg/kg TCDD dose-dependently increased UGT activity for T_4_, but it reduced T_4_ levels in the serum of rats (Craft et al. 2002). Thus, the disruption of thyroid hormone homeostasis by TCDD may be mediated via AhR. Interestingly, TCDD dose-dependently decreased plasma T_4_ levels not only in animals but also in humans (Turyk et al. 2007), although in the latter case, the exposure was evaluated as a dioxin-like toxic equivalent. Other chemicals, such as 3-methyl-cholanthrene (3MC) and polychlorinated biphenyl (PCB) are also known to increase UGT activity for T_4_ (Hood and Klaassen 2000) and to reduce serum T_4_, suggesting that UGT activity is an important factor in the homeostasis of T_4_ levels.

STs structurally have three aromatic rings, suggesting that these chemicals may be AhR agonists and influence UGT activity for T_4_ similar to the action of TCDD, PCB, or 3MC, which also have two or three aromatic rings. To investigate whether STs affect T_4_ levels via AhR-mediated *UGT* genes, we selected ST-1 (CAS no. 18964-53-9; Figure 1) for this study because of its relatively high toxicity: This isomer has higher proliferation activity for MCF-7 and higher affinity for human ER-α than do the other isomers (Ohyama et al. 2007). We investigated whether ST-1 alters T_4_ levels by inducing expression of drug-metabolizing enzymes via AhR similar to that observed with the AhR ligand TCDD. Contrary to expectations, ST-1 down-regulated AhR in a manner distinct from that of the positive activator TCDD.

## Materials and Methods

### Chemicals

We obtained ST-1 (98.9%) from Hayashi Pure Chemical Industry (Osaka, Japan); resorufin, 7-ethoxyresorufin, 1-naphthol, UDP-glucuronic acid, and 3-methyl cholanthrene from Sigma Chemical Co. (St. Louis, MO, USA); anti-AhR goat IgG and biotinlated anti-goat IgG from Santa Cruz Biotechnology Inc. (Santa Cruz, CA, USA); and phosphatase-conjugated streptavidin from Jackson Immuno Research Laboratories Inc. (West Grove, PA, USA).

### Animals

We conducted this animal study according to the Guidelines for Animal Experiments of the Nagoya University Animal Center. The animals were treated humanely and with regard for alleviation of suffering. To clarify the involvement of AhR in the effect of ST-1 on the plasma T_4_ levels, we used two genotyped mice, wild-type and *AhR*-null mice, in this experiment. All mice were housed in cages in a clean room under controlled temperature (23–25°C), relative humidity (57–60%), and light (12/12-hr light/dark cycle). For breeding and for experimental use of wild-type mice, we purchased 8-week-old male mice (C57BL/6N) from CLEA Japan Inc. (Tokyo, Japan). *Ahr*-null mice, described previously by Fernandez-Salguero et al. (1995), were transferred to Nagoya University. *Ahr*-hetero [*Ahr* (+/–)] female mice were mated with *Ahr*-null male mice, and the *Ahr* gene and null allele of their offspring were genotyped. Twelve-week-old wild-type male mice [*Ahr* (+/+)] and *Ahr*-null male mice [*Ahr* (–/–)] were used throughout the experiments.

We treated all mice [eight wild-type mice in the measurement of free triiodothyronine (T_3_) and thyroid-stimulating hormone (TSH); five mice of each genotyped group for the other markers] with 0, 32, and 64 μmol/kg bw ST-1 by gavage for 4 consecutive days. Because no LD_50_ (dose lethal to 50%) has been identified for STs , we determined the dose of ST-1 used in this experiment from the LD_50_ of styrene (3 mmol/kg) [Registry of Cytotoxicity Data (ZEBET) 7.1, National Institutes of Health, Berlin, Germany]. We used 32 μg/kg (low-dose group), which is about 1/100th of the LD_50_ of styrene, and 64 μg/kg (high-dose group), which is 2-fold that of the low-dose group. To determine the exact induction of AhR target genes, we used 3MC as an AhR activator. Twelve-week-old wild-type male mice were given 3MC (20 mg/kg bw) by gavage for 4 consecutive days. All animals were killed by decapitation 16 hr after the last dose, and the blood and livers were quickly removed. A part of the liver was kept for later isolation of total RNA. The remaining liver and the plasma were stored at –80°C until use.

### Real-time quantitative polymerase chain reaction (PCR) analysis

We monitored the mRNA levels of *AhR*, *CYP1A1/2*, *UGT1A1/A6*, *CAR*, and CAR-mediated gene *CYP2B10* on an ABI PRISM 7000 Sequence Detection system (Applied Biosystems, Foster City, CA, USA). Total RNA was extracted from the liver of mice exposed to ST-1, vehicle, or 3MC using the RNeasy Protect Mini Kit (Qiagen, Tokyo, Japan). Complementary DNA (cDNA) was synthesized from 1 μg total RNA using oligo(dT)_20_ primer. RNA quantity and quality were determined using the Gene Quant 2 RNA/DNA calculator (Pharmacia Biotech, Framingham, MA, USA). We designed the primers using Primer Express 1.0 software (Applied Biosystems) (Table 1). The PCR mixture (25 μL) contained 1 × SYBR Green Master Mix (Applied Biosystems), 0.1 μM of forward primer, and cDNA diluted by Tris-EDTA buffer, including 1 mg/mL transfer-RNA (the experiment was repeated for each primer). PCR amplification was as follows: an initial step for 2 min at 50°C, then 10 min at 95°C, followed by 50 cycles at 95°C for 15 sec and 60°C for 1 min. Glyceraldehyde-3-phosphate dehydrogenase (*GAPDH*) was used as a housekeeping gene for data analysis. We normalized all mRNA expression levels to GAPDH mRNA in the same preparation.

### Preparation of microsomes and cytosols

Liver was homogenized with a 3-fold volume of 10 mM phosphate buffer (pH 7.4) containing 0.25 M sucrose. Supernatants were centrifuged at 10,000 × *g* for 10 min, and then further centrifuged at 105,000 × *g* for 60 min. The pellet (microsomal fraction) was resuspended in the same buffer, and the supernatant (cytosol fraction) was stored at –80°C until use.

### Preparation of nuclear fraction

We extracted the nuclear fraction from frozen liver of each mouse using a protein extraction reagent T-PER (Pierce Biotechnology, Rockford, IL, USA).

### Analysis of protein concentrations

We measured protein concentrations of the homogenate, cytosol, and nuclear fractions using Bio-Rad Protein Assay (Bio-Rad, Tokyo, Japan).

### Ethoxyresorufin *O*-deethylase (EROD) activity

We measured EROD activity in liver microsomes using 7-ethoxyresorufin as a substrate according to the method of Hanioka et al. (2000). Liver microsomes (120 μg protein) were mixed in a reaction mixture of 50 mM phosphate buffer (pH 7.4) and 0.1 mM 7-ethoxyresorufin. The reaction was started by the addition of nicotinamide adenine dinucleotide phosphate (NADPH) (10 mM) in a final volume of 500 μL. After incubation at 25°C for 5 min, the reaction was stopped by adding 500 μL ice-cold methanol, and the reaction vial was cooled on ice for 15 min, followed by the centrifugation at 6,000 × *g* for 20 min. The supernatant was filtered with a disposable disk filter (HPLC-DISK-3 0.2 μm; Kanto Kagaku, Tokyo, Japan). Aliquots (10 μL) were subjected to HPLC using a Hitachi L-6000 equipped with an F-1080 fluorescence detector (Hitachi, Tokyo, Japan), an AS-2000 auto sampler, a D-2500 chromatointegrator (Hitachi), and an ODS-80A column (GL Sciences, Tokyo, Japan). As a mobile phase, 20 mM phosphate buffer (pH 6.8):methanol:acetonitrile (52:45:3, vol/vol/vol) was used at a flow rate of 1.0 mL/min. The excitation and emission wavelengths were fixed at 535 nm and 585 nm, respectively.

### UGT activity

We measured UGT activity in liver microsomes using 1-naphthol as a substrate according to the method of Yokota et al. (2002). Liver microsomes (100 μg protein) were treated with 0.25% Triton-X at 37°C for 5 min, followed by adding the reaction mixture [40 mM Tris-HCl buffer (pH 7.4), 0.8 mM MgCl_2_, and 0.2 mM 1-naphthol]. The reaction was started by adding UDP-glucuronic acid (1.8 mM) in a final volume of 500 μL. After the vials were incubated at 37°C for 10 min, the reactions were stopped by the addition of 500 μL ice-cold acetonitrile, and the reaction vial was cooled on ice for 15 min. The mixture was then centrifuged at 10,000 × *g* for 5 min, and the supernatant was filtered with a disposable disk filter (HPLC-DISK-3, 0.2 μm; Kanto Kagaku). HPLC was performed using an aliquot (10 μL) of supernatant and a Hitachi L-6000 equipped with an L-4200 UV-VIS detector, an AS-2000 auto sampler, a D-2500 chromatointegrator (Hitachi), and a GH-C18 column (Hitachi). As a mobile phase, acetonitrile/distilled water/acetic acid (35:65:0.1, vol/vol/vol) was used at a flow rate of 1.0 mL/min. The wavelengths were fixed at 221.5 nm.

### Determination of AhR protein levels by Western blotting

The nuclear fraction (25 μg protein) and the cytosolic fraction (25 μg protein) were separated by 10% SDS-PAGE and transferred to nitrocellulose membranes (Bio-Rad). After blocking with 3% skim milk, each membrane was incubated with anti-AhR antibody (1:200) for 1 hr. The membrane was washed with Tris-buffered saline (TBS, pH 7.4) and TBS containing 0.1% Tween-20 (TBST), and incubated with the biotinlated anti-goat IgG antibody (1:5,000) for 1 hr. After washing the membrane with TBS and TBST, the membrane was incubated with alkaline phosphatase-conjugated streptavidin (1:5,000) for 1 hr. The membrane was washed with TBST and TBS, and specific immune complexes were detected with 1-Step NBT/BCIP (Pierce Biotechnology). Each band was quantified using densitometry with Lane & Spot Analyzer, version 5.0 (ATTO, Tokyo, Japan), and the mean strength of the control group was assigned a value of 1.0.

### Electrophoretic mobility shift assay

A double-stranded xenobiotic response element (*XRE*) fragment corresponding to the consensus *XRE* of *CYP1A1* promoter (forward, 5′-GATCTG-GCTCTTCTCACGCAACTCCG-3′; reverse, 5′-GATCCGGAGTTGCGTGAGAA-GAGCCA-3′) was labeled with biotin using the Biotin-Labeling Kit (Pierce Biotechnology) according to the method of Denison et al. (1988). Nuclear extract (5 μg) was then incubated with biotin-labeled DNA at room temperature for 20 min in 1 × gel shift buffer supplemented with poly(dI-dC; 0.5 μg/μL). The reaction samples were resolved on non-denaturing electrophoresis (4% acrylamide) and transferred to a positively charged nylon membrane (Roche Diagnostics, Mannheim, Germany). AhR–XRE complex was detected with a Chemiluminescent Nucleic Acid Detection Module (Pierce Biotechnology) and visualized using a Lumi Vision PRO HS II (Aisin Seiki, Aichi, Japan). Each band was quantified using densitometry with the Lane & Spot Analyzer, and the mean strength of the control group was assigned a value of 1.0.

### Measurement of thyroid hormone concentration

Total T_4_ concentration in plasma was measured by electrochemiluminescence immunoassay (SRL Inc., Tokyo, Japan). The amounts of free T_4_ in plasma were measured using Amerlex-MAb FT_4_ kits (Trinity Biotech, Bray, Ireland). The levels of free T_3_ in plasma were measured using EIA FREE T3 (T193; Leinco Technologies Inc., St. Louis, MO, USA). Because no kit was available for mouse TSH, we measured the amount of TSH in plasma using a radioimmunoassay kit for rat TSH [National Institute of Diabetes, Digestive, and Kidney Diseases (NIDDK) National Hormone and Peptide Program, National Institutes of Health, Bethesda, MD, USA]. In the preliminary experiment, we confirmed the possibility of the kit’s use for analyzing mouse plasma TSH levels. The antiserum used was anti-rat TSH-S-6 (AFP329691Rb), and the hormone for iodination was rat TSH-I-9 (AFP11542B). Results are expressed in terms of NIDDK rat TSH-RP-3 (AFP5512B) so that we could determine the values relative to those of rats, although the results are not absolute values for mice. The intraassay and interassay coefficients of variation were 3.4% and 5.2%, respectively.

### Statistical analysis

We performed statistical analysis using two-way analysis of variance (ANOVA), followed by Dunnett’s test using JMP, version 4.0 (SAS Institute Inc., Cary, NC, USA). When only wild-type mice were used, Dunnett’s test was employed after one-way ANOVA. *p*-Values < 0.05 were considered statistically significant.

## Results

### ST-1 effects on AhR- and CAR-mediated genes

**Figure 2:**
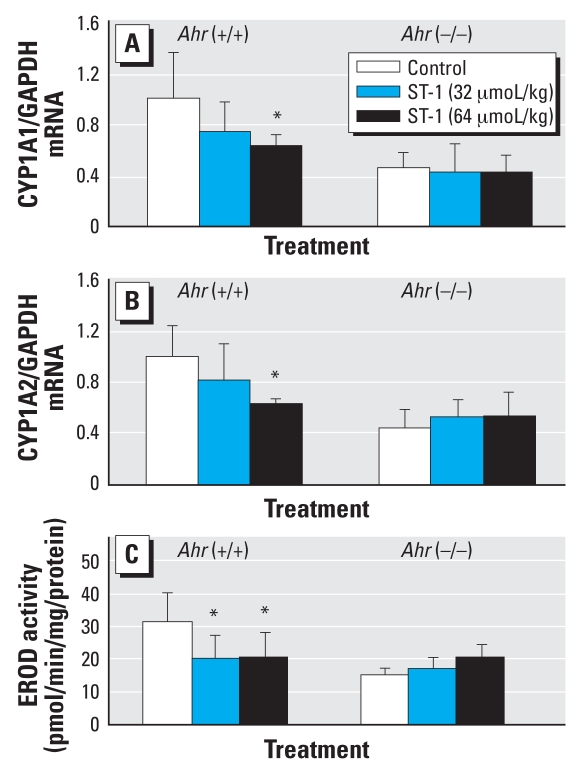
Real-time quantitative PCR analyses of liver CYP1A1 (*A*) and CYP1A2 (*B*) mRNA and microsomal CYP1A enzyme activity (EROD; *C*) in wild-type and *AhR*-null control ST-1–exposed mice. Values represent mean ± SD (*n* = 5). *Significantly different from the corresponding control (*p* < 0.05).

**Figure 3:**
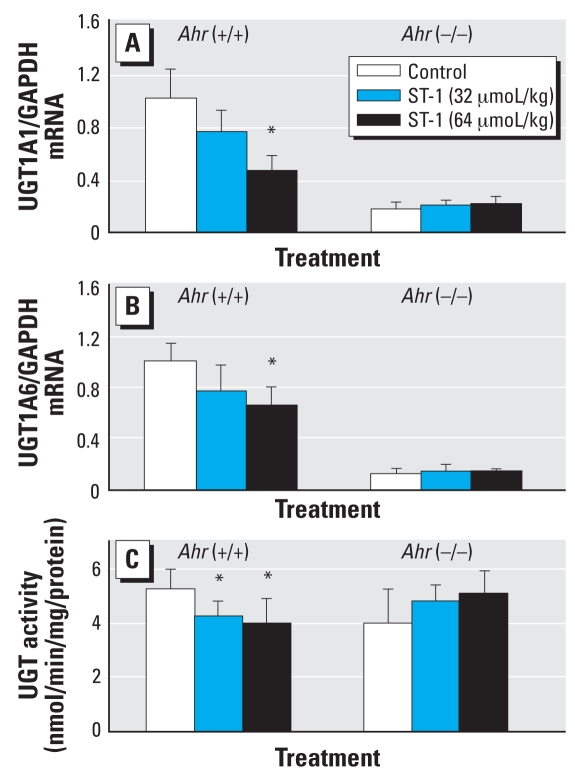
Real-time quantitative PCR analyses of liver UGT1A1 (*A*) and UGT1A6 (*B*) mRNA levels, and microsomal UGT enzyme activity (*C*) in wild-type and *AhR*-null control and ST-1–exposed mice. Values represent mean ± SD (*n* = 5). *Significantly different from the corresponding control (*p* < 0.05).

**Figure 4:**
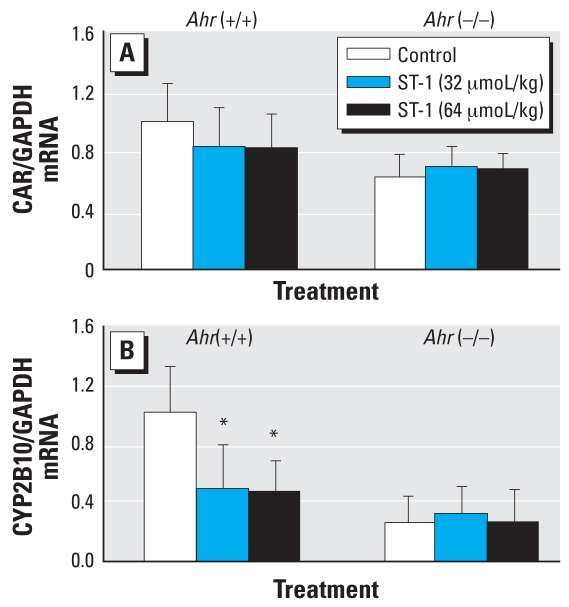
Effects of ST-1 treatments on expression of CAR (*A*) and CYP2B10 (*B*) mRNA levels in the livers of wild-type and *AhR*-null mice. Values represent mean ± SD (*n* = 5). *Significantly different from the corresponding control (*p* < 0.05).

ST-1 treatment did not influence the body or organ weights of wild-type and *AhR*-null mice (data not shown). To determine the up- or down-regulation of AhR by ST-1, we measured *CYP1A1* and *CYP1A2* genes and their activity. High-dose (64 μmol/kg) ST-1 significantly decreased the expressions of CYP1A1 and CYP1A2 mRNAs in the livers of wild-type mice but did not influence those in *Ahr*-null mice (Figure 2A,B). Similarly, high and low (32 μmol/kg) dosages of ST-1 significantly decreased CYP1A1 activity, as measured by the rate of dealkylation of 7-ethoxyresorufin, only in the liver microsomes from wild-type mice (Figure 2C). High-dose ST-1 also significantly decreased the expressions of UGT1A1 and UGT1A6 mRNAs only in the liver from wild-type mice (Figure 3A,B). Similarly, high- and low-dose ST-1 significantly decreased UGT activity for 1-naphthol as a substrate in liver microsomes from wild-type mice but not in *Ahr*-null mice (Figure 3C). High- and low-dose ST-1 also significantly decreased the expression of CYP2B10 mRNA in the liver from wild-type mice, but not in that from *Ahr*-null mice. ST-1 did not alter CAR mRNA (Figure 4).

### ST-1 effects on expression of AhR mRNA and protein

**Figure 5:**
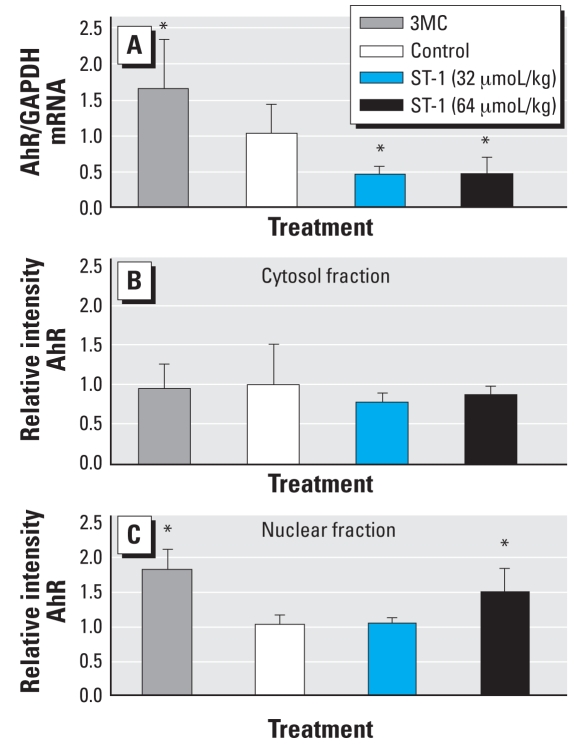
Real-time quantitative PCR analysis of AhR mRNA in the liver (*A*) and immunoblot analysis AhR protein in the cytosol (*B*) and nuclear (*C*) fractions of control, 3MC-, and ST-1–exposed wild-type mice. Liver samples from 3MC-treated mice were used to determine the induction of AhR and AhR-target genes. Values represent mean ± SD (*n* = 5). *Significantly different from the corresponding control by Dunnett’s test (*p* < 0.05).

Because ST-1 treatment significantly decreased the CYP1A1 and CYP1A2 mRNA levels only in wild-type mouse liver, we measured the expressions of AhR mRNA and protein in wild-type mouse liver. As a positive marker for the induction of AhR, we also used the livers of wild-type mice treated with 3MC. 3MC significantly induced AhR mRNA in the liver and the protein in the nuclear fraction (Figure 5A,C). In contrast, ST-1 treatment significantly decreased the expression of AhR mRNA. ST-1 treatments unexpectedly increased AhR protein in the hepatic nucleus. 3MC and ST-1 did not influence the AhR protein levels in the cytosol fractions (Figure 5B).

### Electrophoretic mobility shift assay

**Figure 6:**
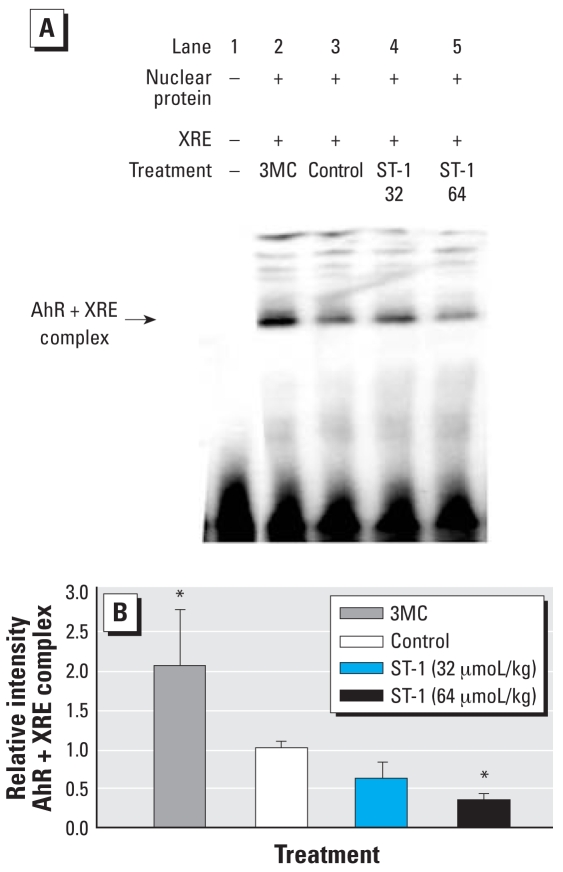
Electrophoresis mobility shift assay of AhR–XRE complex in liver nuclear fraction of control, 3MC-treated, and ST-1–treated wild-type mice (*A*) and quantification of the assay by densitometric analysis (*B*), with the mean strength of the control group assigned a value of 1.0. Values represent the mean ± SD (*n* = 5). *Significantly different from the correspondng control by Dunnett’s test (*p* < 0.05).

Because ST-1 treatment significantly decreased the AhR, CYP1A1, and CYP1A2 mRNA levels but AhR protein accumulated in hepatic nuclei from wild-type mice, we analyzed expression of AhR binding to *XRE* by electrophoretic mobility shift assay. As a positive marker, we also used nuclear protein of mice treated with 3MC. AhR–XRE complexes were detected in all nuclear samples. 3MC significantly increased the expression of AhR–XRE complexes, but high-dose ST-1 decreased the expression compared with that of the hepatic nucleus of controls (Figure 6).

### ST-1 effects on thyroid hormone

Because ST-1 treatment significantly decreased the expressions of UGT1A1 and UGT1A6 mRNA as well as activity, we measured T_4_ levels in the plasma in wild-type and *Ahr*-null mice. We detected no differences in the plasma total T_4_ levels between control groups of wild-type and *Ahr*-null mice (Table 2). High-dose ST-1 increased the total T_4_ level in the plasma from wild-type mice but not from *Ahr*-null mice. Therefore, we measured plasma free T_4_, free T_3_, and TSH levels only in wild-type mice. High-dose ST-1 also increased free T_4_ levels but did not influence T_3_ and TSH levels.

## Discussion

The present study revealed that ST-1 exposure decreased expression of the *CYP1A1*, *CYP1A2*, *UGT1A1*, and *UGT1A6* genes via suppression of the *AhR* gene in mice, as shown by the decrease of mRNAs and activity in the wild-type mice, but not in *Ahr*-null mice. On the contrary, AhR protein accumulated in the nucleus but AhR-XRE complex decreased in the nucleus of ST-1–treated mouse liver. These findings are considerably different from the transcriptional activation of AhR by TCDD, PCB, or 3MC, which significantly induce the drug-metabolizing enzymes described above via AhR and increase AhR protein and AhR-XRE complex in the nucleus (Gonzalez 1998; Shimizu et al. 2000). Although it is unclear why the expression of AhR protein in the nucleus stained by anti-AhR was increased by ST-1 treatment, high-dose ST-1 increased the plasma T_4_ level only in the wild-type mice, which may have been due in part to the AhR down-regulation by ST-1 and consequent decrease in UGT1A expression. To our knowledge, this is the first study to demonstrate that a relatively high dose of ST-1 induced an increase in T_4_ plasma levels in mice.

T_4_ is conjugated with UDP-glucuronic acid by the catalytic action of UGT1A1 and UGT1A6, which have XRE in the promoter region and are activated by AhR agonists such as TCDD, PCB, and 3MC in Wistar rats (Emi et al. 1995). In fact, Nishimura et al. (2005) reported that TCDD markedly reduced serum total T_4_ and free T_4_ levels only in *AhR* heterozygous mice by inducing UGT1A6 mRNA and thereby increased biliary excretion of the T_4_ glucuronide. Unfortunately, in their study, they did not measure whether TCDD also induced UGT1A1 only in the liver. On the other hand, 3MC and phenobarbital, which are AhR and CAR agonists, respectively, induced UGT1A1 mRNA and protein (Emi et al. 1996; Mackenzie et al. 1997; Sugatani et al. 2001, 2004). Because *CYP2B10* is a CAR target gene (Maglich et al. 2004; Wei et al. 2000), the decreased level of CYP2B10 mRNA by ST-1 treatment suggests that this compound might also down-regulate CAR and participate in the decreased expression of UGT1A1, although the expression of CAR mRNA did not decrease significantly. Taken together, CAR may also be involved, in part, in the decrease in UGT1A1 by ST-1 treatment. However, we cannot rule out the involvement of CAR in the expression of UGT1A6 in the present study. Thus, ST-1 treatment down-regulated *UGT1A1* and *UGT1A6* genes, which might result in increased total T_4_ and free T_4_ levels in serum because of the decrease in the conjugation of T_4_ with UDP-glucuronide and a decrease in the excretion of T_4_ to the bile duct. The influence of ST-1 on the plasma total T_4_ and free T_4_ levels was contrary to that of TCDD or 3MC. In contrast to T_4_ levels, plasma T_3_ levels were not influenced by ST-1 treatment. 3MC or PCB treatment for 7 days (Hood and Klaassen 2000; Vansell and Klaassen 2001) and a single oral administration of TCDD (Nishimura et al., 2002) also did not influence plasma T_3_ levels in rats, although these chemicals decreased T_4_ levels in plasma, suggesting that T_3_ levels would not be influenced by such chemicals.

Although relatively short-term exposure (7 days) to 3MC and PCB decreased T_4_ levels in plasma in rats, they did not influence plasma TSH levels (Hood and Klaassen 2000). In contrast, long-term exposure (8 weeks) to one of the catechins involved in green tea (polyphenone-60) decreased T_4_ levels and, conversely, increased TSH levels (Satoh et al. 2002). In our experiment, ST-1 treatment did not decrease TSH levels in the plasma of wild-type mice, although it did increase T_4_ levels. It is unknown whether the unchanged TSH is due to the short-term exposure to ST-1. Because the effect of ST-1 treatment on plasma T_4_ levels was investigated only from the viewpoint of T_4_ glucuronidation, the effects on thyroid hormone biosynthesis, thyroid hormone signaling in thyroid, and TSH biosynthesis and release in hypothalamus and pituitary were not investigated. Further study is warranted to determine whether ST-1 treatment influences this process. Of course, the effect of long-term exposure to ST-1 on thyroid hormone systems also has to be determined. In fact, certain catechins involved in green tea—which down-regulate the AhR-related gene *CYP1A1* and also might be suspected to down-regulate UGT1A—caused histopathologic changes in thyroid (Goodin et al. 2006; Sakamoto et al. 2001).

A model for the nucleocytoplasmic shuttling of AhR has been reported (Ikuta et al. 2000; Pollenz and Barbour 2000). First, some ligands bind to AhR associated with heat-shock protein 90 (HSP90), which translocates into the nucleus. In the nucleus, AhR and AhR nuclear translocator (Arnt) dimerize to form the AhR–Arnt heterodimer, which is competent to bind *XRE* in the promoter of specific genes such as *CYP1A1*. After transactivating the target gene, the heterodimer is dissociated by the action of the nuclear export signal (NES). Chromosome region maintenance 1 protein (CRM1), which participates in nuclear protein export as a receptor of NES, accesses and recognizes the NES. Then, AhR bound to CRM1 is exported from the nucleus to the cytoplasm. In general, the AhR agonist induces AhR-mediated genes such as *CYP1A1*, but the antagonist leptomycin B was found to inhibit gene induction (Ikuta et al. 2000; Pollenz and Barbour 2000). In the latter case, AhR protein recognized by anti-AhR accumulated in the nucleus. Because leptomycin B inhibited AhR export, the inactive AhR remained in the nucleus and therefore down-regulated transcription of AhR-mediated genes (Ikuta et al. 2000; Pollenz and Barbour 2000). In the present study, ST-1 treatment increased AhR recognized by anti-AhR. Thus, ST-1 might inhibit AhR export, resulting in accumulation of the inactive AhR in the nucleus, similar to that of leptomycin B. Taken together, ST-1 might down-regulate the AhR-mediated genes by both down-regulation of *AhR* gene and the inhibition of export of inactive AhR from the nucleus into cytosol.

## Conclusion

The high dose of ST-1 used in this study significantly down-regulated AhR and the mediated genes such as *UGT1A1/A6*, which may have resulted, in part, in the increase in plasma T_4_ levels. Although this dose is considerably higher than levels of ST-1 in the general environment, this new information is very valuable for the risk assessment of STs.

## Figures and Tables

**Figure 1:**
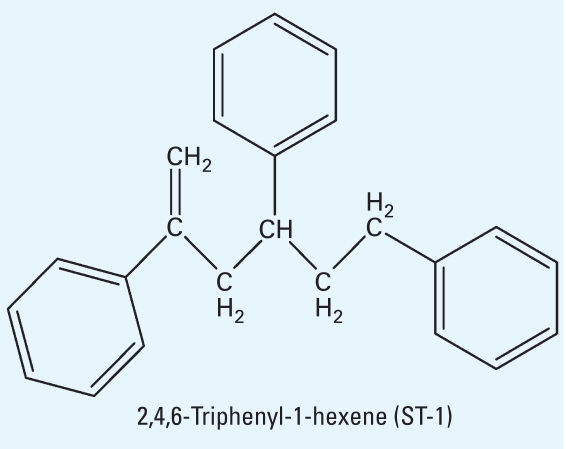
Chemical structure of ST-1.

**Table 1:** Primers used for real-time PCR to quantify mRNA expression of AhR-related gene.

Gene (GenBank accession number)^a^	Forward primer (5′ to 3′)	Reverse primer (5′ to 3′)
*AhR* (NM 013464)	CTGTGTCCTTCAAGC	CCGCACTTGCTCACGGA
*CYP1A1* (NM009992)	CGTTCCAGCTTCCTGTCCTGA	AGGACATTTGAGAAGGGCCA
*CYP1A2* (NM009993)	CCATGTGCTTTGGGAAGAACTT	GTCCTTGCTGTTATTCACGATGTT
*UGT1A1* (AF093878)	TGAACTTCCTACAGCGACTGAAGA	GGGAATAAACCACTCTGCACATAA
*UGT1A6* (D87867)	CCTTTGGAAACAACCACTTTCTTC	CGACAATCATGTTGTTCCTGTACTC
*CAR* (AF009327)	CAGGGTCCAGTACGAGTTTTTG	AGGCTCCTGGAGATGCAGTC
*CYP2B10* (AF128849)	CCAACCAGCACACGGACTT	CGGTGCCAGCAAAGAAGAG
*GAPDH* (BC096440)	AGAACATCATCCCTGCATCCA	CCGTTCAGCTCTGGGATGAC

a GenBank data are available from the National Center for Biotechnology Information (2008).

**Table 2:** Plasma thyroid hormone levels (mean ± SD) in wild-type and *AhR*-null type mice.

				ST-1 (μmol/kg)	
Hormone	Mice	No.	Control	32	64
Total T_4_ (ng/mL)	*AhR* (+/+)	5	31.8 ± 4.9	47.9 ± 13.3	55.3 ± 11.7*
Total T_4_ (ng/mL)	*AhR* (−/−)	5	31.0 ± 7.7	39.7 ± 4.6	40.4 ± 6.4
Free T_4_ (pg/mL)	*AhR* (+/+)	5	5.7 ± 1.8	6.7 ± 8.8	28.5 ± 13.9*
Free T_3_ (pg/mL)	*AhR* (+/+)	8	1.0 ± 0.3	1.7 ± 0.2	1.3 ± 0.2
TSH (pg/mL)	*AhR* (+/+)	8	300 ± 213	305 ± 301	286 ± 187

* Significantly different from the control group (*p* < 0.05).

## References

[b1-ehp0116-000740] Bachmann S, Hellwig J, Jackh R, Christian MS (1998). Uterotrophic assay of two concentrations of migrates from each of 23 polystyrenes administered orally (by gavage) to immature female Wistar rats. Drug Chem Toxicol.

[b2-ehp0116-000740] Burchell B, Coughtrie MW (1997). Genetic and environmental factors associated with variation of human xenobiotic glucuronidation and sulfation. Environ Health Perspect.

[b3-ehp0116-000740] Craft SC, DeVito MJ, Crofton KM (2002). Comparative responsiveness of hypothyroxinemia and hepatic enzyme induction in Long-Evans rats versus C57BL/6J mice exposed to TCDD-like and phenobarbital-like polychlorinated biphenyl congeners. Toxicol Sci.

[b4-ehp0116-000740] Date K, Ohno K, Azuma Y, Hirano S, Kobayashi K, Sakurai T (2002). Endocrine-disrupting effects of styrene oligomers that migrated from polystyrene containers into food. Food Chem Toxicol.

[b5-ehp0116-000740] Denison MS, Fisher JM, Whitlock JP (1988). The DNA recognition site for the dioxin-Ah receptor complex. Nucleotide sequence and functional analysis. J Biol Chem.

[b6-ehp0116-000740] Emi Y, Ikushiro S, Iyanagi T (1995). Drug-responsive and tissue-specific alternative expression of multiple first exons in rat UDP-glucuronosyltransferase family (UGT1) gene complex. J Biochem (Tokyo).

[b7-ehp0116-000740] Emi Y, Ikushiro S, Iyanagi T (1996). Xenobiotic responsive element-mediated transcriptional activation in the UDP-glucuronosyltransferase family gene complex. J Biol Chem.

[b8-ehp0116-000740] Fernandez-Salguero P, Pineau T, Hibert DM, McPhail T, Lee SS, Kimura S (1995). Immune system impairment and hepatic fibrosis in mice lacking the dioxin-binding Ah receptor. Science.

[b9-ehp0116-000740] Gonzalez FJ (1998). The study of xenobiotic-metabolizing enzymes and their role in toxicity in vivo using targeted gene disruption. Toxicol Lett.

[b10-ehp0116-000740] Goodin MG, Bray BJ, Rosengren RJ (2006). Sex- and strain-dependent effects of epigallocatechin gallate (EGCG) and epicatechin gallate (ECG) in the mouse. Food Chem Toxicol.

[b11-ehp0116-000740] Hanioka N, Tatarazako N, Jinni H, Arizono K, Ando M (2000). Determination of cytochrome P450 1A activities in mammalian liver microsomes by high-performance liquid chromatography with fluorescence detection. J Chromatogr B Biomed Sci Appl.

[b12-ehp0116-000740] Hood A, Klaassen CD (2000). Differential effects of microsomal enzyme inducers on in vitro thyroxine (T_4_) and triiodothyronine (T_3_) glucuronidation. Toxicol Sci.

[b13-ehp0116-000740] Ikuta T, Tachibana T, Watanabe J, Yoshida M, Yoneda Y, Kawajiri K (2000). Nucleocytoplasmic shuttling of the aryl hydrocarbon receptor. J Biochem (Tokyo).

[b14-ehp0116-000740] Kaneko R, Funayama K, Haneishi N, Watanabe Y, Ogino S (2003). Survey of amount of styrene dimers and trimers in instant-noodles contained in polystyrene cups [in Japanese]. Ann Rep Tokyo Metr Inst PH.

[b15-ehp0116-000740] Kawamura Y, Nishi K, Maehara T, Yamada T (1998). Migration of styrene dimer and trimers from polystyrene containers into instant foods. J Food Hyg Soc Japan.

[b16-ehp0116-000740] Mackenzie PI, Owens IS, Burchell B, Bock KW, Bairoch A, Belanger A (1997). The UDP glycosyltransferase gene superfamily: recommended nomenclature update based on evolutionary divergence. Pharmacogenetics.

[b17-ehp0116-000740] Maglich JM, Watson J, McMillen PJ, Goodwin B, Wilson TM, Moore JT (2004). CAR is a regulator of thyroid hormone metabolism during caloric restriction. J Biol Chem.

[b18-ehp0116-000740] National Center for Biotechnology Information (2008). Genbank Overview. http://www.ncbi.nlm.nih.gov/Genbank.

[b19-ehp0116-000740] Nishimura N, Miyabara Y, Sato M, Yonemoto J, Tohyama C (2002). Immunohistochemical localization of thyroid stimulating hormone induced by a low oral dose of 2,3,7,8-tetra-chlorodibenzo-*p*-dioxin in female Sprague-Dawley rats. Toxicology.

[b20-ehp0116-000740] Nishimura N, Yonemoto J, Miyabara Y, Fujii-Kuriyama Y, Tohyama C (2005). Altered thyroxin and retinoid metabolic response to 2,3,7,8-tetrachlorodibenzo-*p*-dioxin in aryl hydrocarbon receptor-null mice. Arch Toxicol.

[b21-ehp0116-000740] Nobuhara Y, Hirano S, Azuma Y, Date K, Ohno K, Tanaka K (1999). Biological evaluation of styrene oligomers for endocrine-disrupting effects. J Food Hyg Soc Japan.

[b22-ehp0116-000740] Ohno K, Azuma Y, Date K, Nakano S, Kobayashi T, Nagao Y (2002a). Estrogenicity of styrene oligomers and assessment of estrogen receptor binding assays [Letter]. Environ Health Perspect.

[b23-ehp0116-000740] Ohno K, Azuma Y, Nakano S, Kobayashi T, Hirano S, Nobuhara Y (2002b). Assessment of styrene oligomers eluted from polystyrene-made food containers for estrogenic effects in in vitro assays. Food Chem Toxicol.

[b24-ehp0116-000740] Ohyama K, Nagai F, Tsuchiya Y (2001). Certain styrene oligomers have proliferative activity on MCF-7 human breast tumor cells and binding affinity for human estrogen receptor. Environ Health Perspect.

[b25-ehp0116-000740] Ohyama K, Satoh K, Sakamoto Y, Ogata A, Nagai F (2007). Effects of prenatal exposure to styrene trimers on genital organs and hormones in male rat. Exp Biol Med (Maywood).

[b26-ehp0116-000740] Pollenz RS, Barbour ER (2000). Analysis of the complex relationship between nuclear export and aryl hydrocarbon receptor-mediated gene regulation. Mol Cell Biol.

[b27-ehp0116-000740] Sakamoto H, Matsuzawa A, Itoh R, Tohyama Y (2000). Quantitative analysis of styrene dimer and trimers migrated from disposable lunch boxes. J Food Hyg Soc Japan.

[b28-ehp0116-000740] Sakamoto Y, Mikuriya H, Tayama K, Takahashi H, Yano N, Aoki N (2001). Goitrogenic effects of green tea extract catechins by dietary administration in rats. Arch Toxicol.

[b29-ehp0116-000740] Satoh K, Nagai F, Aoki N (2001). Several environmental pollutants have binding affinities for both androgen receptor and estrogen receptor a. J Health Sci.

[b30-ehp0116-000740] Satoh K, Sakamoto Y, Ogata A, Nagai F, Mikuriya H, Aoki N (2002). Inhibition of aromatase activity by green tea extract catechins and their endocrinological effects of oral administration in rats. Food Chem Toxicol.

[b31-ehp0116-000740] Shimizu Y, Nakatsuru Y, Ichinose M, Takahashi Y, Kume H, Mimura J (2000). Benzo(*a*)pyrene carcinogenicity is lost in mice lacking the aryl hydrocarbon receptor. Proc Natl Acad Sci USA.

[b32-ehp0116-000740] Sugatani J, Kojima H, Ueda A, Kakizaki S, Yoshinari K, Gong QH (2001). The phenobarbital response enhancer module in the human bilirubin UDP-glucuronosyltransferase UGT1A1 gene and regulation by the nuclear receptor CAR. Hepatology.

[b33-ehp0116-000740] Sugatani J, Yamakawa K, Tonda E, Nishitani S, Yoshinari K, Degawa M (2004). The induction of human UDP-glucuronosyltransferase 1A1 mediated through a distal enhancer module by flavonoids and xenobiotics. Biochem Pharmacol.

[b34-ehp0116-000740] Turyk ME, Anderson HA, Persky VW (2007). Relationships of thyroid hormones with polychlorinated biphenyls, dioxins, furans, and DDE in adults. Environ Health Perspect.

[b35-ehp0116-000740] Vansell NR, Klaassen CD (2001). Increased biliary excretion of thyroxine by microsomal enzyme inducers. Toxicol Appl Pharmacol.

[b36-ehp0116-000740] Wei P, Zhang J, Egan-Hafley M, Liang S, Moore DD (2000). The nuclear receptor CAR mediates specific xenobiotic induction of drug metabolism. Nature.

[b37-ehp0116-000740] Yokota H, Kunimasa Y, Shimoyama Y, Kobayashi T, Matsumoto J, Yuasa A (2002). Effects on extrahepatic UDP-glucuronosyltransferase in hypophysectomized rat. J Biochem (Tokyo).

[b38-ehp0116-000740] Zhou J, Zhang J, Xei W (2005). Xenobiotic nuclear receptor-mediated regulation of UDP-glucuronosyltransferase. Curr Drug Metab.

